# Subconjunctival Injection of Regulatory T Cells Potentiates Corneal Healing Via Orchestrating Inflammation and Tissue Repair After Acute Alkali Burn

**DOI:** 10.1167/iovs.61.14.22

**Published:** 2020-12-16

**Authors:** Dan Yan, Fei Yu, Liangbo Chen, Qinke Yao, Chenxi Yan, Siyi Zhang, Nianxuan Wu, Danni Gong, Hao Sun, Yao Fu, Chunyi Shao

**Affiliations:** 1 Department of Ophthalmology, Ninth People's Hospital, Shanghai JiaoTong University School of Medicine, Shanghai, China; 2 Shanghai Key Laboratory of Orbital Diseases and Ocular Oncology, Shanghai, China

**Keywords:** corneal alkali burn, regulatory T cells, amphiregulin

## Abstract

**Purpose:**

This study aimed to investigate the therapeutic effects and underlying mechanisms of locally delivered regulatory T cells (Tregs) on acute corneal wound healing after alkali burn.

**Methods:**

After corneal alkali burn, the mice were injected subconjunctivally with regulatory T cells (Tregs) isolated from syngeneic mice. The wound healing process was monitored by clinical manifestation, flow cytometry, and enzyme-linked immunosorbent assay (ELISA). As amphiregulin (Areg) was significantly upregulated, its reparative function in injured corneas was suggested. The hypothesis was further verified via loss- and gain-of-function experiments by administrating the antibody of Areg (anti-Areg) and recombinant Areg (rmAreg).

**Results:**

Subconjunctivally injected Tregs rapidly migrated to injured corneas. The mice treated with Tregs showed prominently reduced corneal opacity, alleviated edema, and faster re-epithelialization compared with the control group. Mechanistically, Treg treatment led to suppressed infiltration of inflammatory cells, along with improved proliferation and inhibited apoptosis of corneal epithelial cells. Tregs expressed upregulated functional markers, including Areg. Expectantly, the levels of Areg in corneas were dramatically higher in the Treg injection group, in line with better corneal restoration. Additional experiments showed that the administration of anti-Areg blunted the reparative effect of Tregs, while exogenous Areg enhanced it. Treg-treated corneas also exhibited less neovascularization and fibrosis at a later reconstruction stage of corneal repair.

**Conclusions:**

The findings showed that the subconjunctival injection of Tregs effectively promoted corneal wound healing by inhibiting excessive inflammation and enhancing epithelial regeneration, with an indispensable reparative role of Areg. Subsequent complications of corneal vascularization and fibrosis were therefore reduced.

Corneal chemical injuries are responsible for 11.5% to 22.1% of ocular emergencies.^1^ Among them, corneal alkali burns are usually more severe than other types of injuries because alkali reagents can rapidly penetrate tissues due to their lipophilic properties.^2^ Thus, prompt treatment is necessary to deal with such cases. Despite supportive medical management, treatments, such as topical antibiotics, steroids, and autologous serum, are elusive in controlling inflammation in an acute stage.^3^ Subsequent corneal scar formation and neovascularization occur during wound healing, which are the major complications causing permanent blindness after injuries.^4^ Therefore, effective treatments need to be developed to promote corneal restoration in initial stages and improve prognosis at a later reconstruction period to preserve visions.

Corneal alkali burn is characterized by an overwhelmingly inflammatory response triggered by the infiltration of leukocytes along with excess pro-inflammatory cytokines. An imbalance between anti- and pro-inflammatory response induces excessive inflammation.^5^ Meanwhile, corneal keratolysis always occurs due to excessive inflammation and impaired re-epithelialization.^6^ Thus, strategies for treating acute ocular alkali burns aim at reducing inflammation and accelerating re-epithelization to prevent complications.^7^ Foxp3-expressing regulatory T cells (Tregs) are a distinct population of lymphocytes known for maintaining immune quiescence by muffling the activation of proinflammatory genes, blunting the recruitment of inflammatory T cells, and curtailing inflammatory positive-feedback loops.^8^ The use of Treg-based immunotherapies in treating autoimmune and inflammatory diseases has been widely investigated.^9^ Apart from their remarkable immunoregulatory capacity, the potential of Tregs directly resolving damages by proliferating cells during tissue healing has recently been uncovered in muscle regeneration, hair follicle cycling, and so on.^10^^,^^11^ Therefore, this study focused on the therapeutic effect of Tregs in treating corneal alkali burns.

Accumulated Tregs were detected in corneas shortly after acute alkali injury. Based on a previous study highlighting that Tregs injected subconjunctivally could migrate to corneas and exert their function in corneal transplantation,^12^ we used the same approach to magnify the potential impact of this population in treating severe corneal alkali burns. As expected, a sufficient number of adoptive Tregs reached injured corneas. Corneas treated by Tregs not only showed a decreased inflammatory response but also presented accelerated re-epithelization along with improved prognosis of reduced corneal fibrosis and neovascularization. The functional markers of Tregs were examined to further identify the activated state of Tregs, which revealed a significant increase in amphiregulin (Areg) expression. After gain- and loss-of-function experiments on the functions of Areg, recombinant Areg (rmAreg) was found to accelerate wound healing and the antibody of Areg (anti-Areg) could neutralize the improvement in Treg-induced wound repair, indicating an indispensable role of Areg in the efficacy of Tregs in potentiating tissue repair.

The findings showed that the local delivery of Tregs could be a novel and promising strategy for treating corneal alkali burns through suppression of excessive inflammatory response and promotion of corneal healing via the upregulation of Areg.

## Materials and Methods

### Corneal Alkali-Burn Model

All experiments with mice were approved by the Medical Ethics Committee of Ninth People's Hospital, Shanghai Jiao Tong University School of Medicine. Eight-week-old male BALB/c mice were purchased and housed in a pathogen-free environment from the Animal Center of Ninth People's Hospital, Shanghai Jiao Tong University School of Medicine. Corneal structure, stiffness, and function have been found to be different between men and women, and sex has been considered as one of the important factors that can affect wound healing process. Particularly, the estrogen has been shown to promote the function of immune cells and cytokine synthesis.^13^ An epidemiological human study showed that men have a higher acquisition rate of ocular trauma than women.^14^ In order to exclude the potential influence of estrogen on wound healing, we used male mice in our study, which we believe could be more representative and suitable to investigate the role of regulatory T cells in corneal alkali burn. After anesthetizing mice and ocular administration of oxybuprocaine hydrochloride eye drops (Santen Pharmaceutical Co., Ltd., Kita-ku, Osaka, Japan), a unilateral alkali-burn model was created on the right eye. Then, a 2-mm-diameter Whatman III filter paper disk presoaked with 1N NaOH (Sigma–Aldrich, St. Louis, MO, USA) for 10 seconds was placed on the central cornea for 40 seconds. Extensive rinsing with phosphate-buffered saline (PBS) (Sigma–Aldrich) was conducted for 1 minute. Eyes with complications of hemorrhage, cataract, or infection were excluded from the analysis. Mice were euthanized via carbon dioxide asphyxiation for 5 minutes, followed by cervical dislocation. Minimization of suffering was prioritized.

### Cornea and Conjunctiva Digestion

Corneas and conjunctiva were excised and digested in RPMI medium (Lonza, Rockville, MD, USA), which contained 1.5 mg/mL DNase I (Roche, Mannheim, Germany) and 2 mg/mL collagenase D (Roche, Mannheim, Germany) for 60 minutes at 37°C. Afterwards, the tissues were filtered through a 70-µm cell strainer (Corning Inc., Corning, NY, USA).

### Isolation of Tregs

According to protocols of mouse Treg cell isolation kits (Miltenyi Biotec, Bergisch-Gladbach, Germany), CD4^+^CD25^+^ Tregs were isolated from secondary lymph nodes and spleens of naïve BALB/c mice by magnetic-assisted cell sorting. The purity of Foxp3^+^ Tregs among sorted cells was >85%, as examined by flow cytometry analysis (data not shown).

### Subconjunctival Injection of Tregs

Further, 1 × 10^5^ Treg cells were suspended in 10 µL of PBS and were injected subconjunctivally to alkali-injured mice using a 33-gauge metal needle and a 50-µL syringe (Hamilton Company, Reno, NV, USA). Mice receiving 10 µL of PBS served as the control. In in vivo tracing of the dynamic migration of Tregs, 5(6)-carboxyfluorescein diacetate succinimidyl ester (CFDA-SE) (Sigma Aldrich, St. Louis, MO, USA) was used to stain the sorted Tregs before the injection. According to the protocol of CFDA-SE, the cells were incubated with the dye in the dark at 37°C for 12 minutes, followed by the addition of an equal volume of the culture medium containing 10% fetal bovine serum (Invitrogen) for 5 minutes to quench the excessive dye.

### Flow Cytometry

Each group consisted of 3 samples, and every sample contained 4 ipsilateral corneas. Pooled corneas and conjunctiva were used for flow cytometry. All experiments were performed with three repeats. Fc receptor–blocking antibody (R&D Systems, Minneapolis, MN, USA) was used to incubate cell suspension for 10 minutes to avoid nonspecific staining. The cells were subsequently stained with the fluorescence-conjugated antibodies against CD4 (Peprotech), CD25 (Peprotech), Foxp3 (Peprotech), CD45 (Peprotech), F4/80 (Peprotech), CD86 (Peprotech), CD206 (Peprotech), and Areg (Biorbyt). Proper isotype controls were applied for every antibody. Foxp3/transcription factor staining buffer set (Invitrogen) was used for Foxp3 staining. The cells were stained according to the protocol of the set. An LSRII flow cytometer (BD Biosciences) was used to analyze stained cells. The results were analyzed using FlowJo software (FlowJo LLC, Ashland, OR, USA).

### Clinical Evaluation

Certain examinations for mice from PBS and Treg groups were conducted at indicated time. Data were combined from 3 independent experiments (each group contained 12 mice at one experiment).

### Ocular Dye Vital Staining and Clinical Scoring

Corneal epithelial defects were examined using 2% fluorescein sodium (Sigma–Aldrich) staining under cobalt blue light. Photographs were taken using a slit-lamp microscope (SL-D7; Topcon Inc., Tokyo, Japan) and further processed with Image-Pro Plus V6.0 (Media Cybernetics, Rockville, MD, USA) to calculate defect areas. In the late stage after alkali injury, corneal fluorescein staining was evaluated according to the National Eye Institute/Industry (NEI) grading scale in a blinded manner by two professional ophthalmologists. The scores of corneal epithelial defects were recorded.

### Assessment of Corneal Thickness Using Ultrahigh-Resolution Optical Coherence Tomography

Optical coherence tomography (OCT; Heidelberg Engineering, Germany) was used to measure the central corneas of alkali-injured mice thrice for each eye. The average of three readings was taken at each time point. The thicknesses of corneas were recorded and further analyzed.

### Examination of Corneal Opacity and Corneal Neovascularization Using a Slit-Lamp Microscope

Corneal opacity was examined using a slit-lamp microscope (SL-D7; Topcon Inc.). A standardized opacity and corneal neovascularization grading system was used to define the repair. Corneal opacity was scored on a scale of 0 to 4: 0, completely clear; 1, slightly hazy, and the iris and pupil easily visible; 2, slightly opaque, and the iris and pupil still detectable; 3, opaque, and pupils hardly detectable; and 4, completely opaque with no view of the pupil. Neovascularization was scored on a scale of 0 to 3: 0, no neo-vessels; 1, neo-vessels at the corneal limbus; 2, neo-vessels spanning the corneal limbus and approaching the corneal center; and 3, neo-vessels spanning the corneal center.

### Histological Examination

The mice were ethically sacrific at selected time points. Enucleated eyes were fixed in 10% formaldehyde and paraffin (Sigma–Aldrich) embedded. Serial 4-µm-thick sections were cut and stained with hematoxylin/eosin or Masson's Goldner (MG). Immunofluorescent staining was performed in frozen tissue sections. The sections were fixed for 1 hour at room temperature in 4% paraformaldehyde. They were rinsed three times for 15 minutes each in PBS with 0.3% Triton X-100 (Sigma–Aldrich) and blocked at room temperature for 2 hours in PBS–Triton X-100 containing 5% bovine serum albumin (Sigma–Aldrich). After rinsing three times with PBS, the frozen sections were incubated with antibodies of NF-κB (1:100, Invitrogen), NLRP3 (1:100, Abcam), caspase-1 (1:50, Abcam), IL-1β (1:100, Invitrogen), CK12 (1:100, Invitrogen), ZO-1 (1:50, Invitrogen), PAX 6 (1:100, Abcam), Ki67 (1:50, Abcam), p-Akt (1:100, Abcam), p-ERK (1:100, Abcam), caspase-3 (1:100, Invitrogen), Bcl (1:200, Invitrogen), Areg (1:100, Abcam), α-SMA (1:100, Invitrogen), collagen III (1:200, Invitrogen), and VEGF-A (1:100, Abcam) overnight at 4°C. The following day, the slides were rinsed three times with the washing buffer and incubated with Alexa Fluor-conjugated secondary antibodies (1:400, Life Technologies). After extensive washing, nuclei were further stained with DAPI (Invitrogen). Terminal deoxynucleotidyl transferase dUTP nick-end labeling (TUNEL) staining was performed using an ApopTag plus fluorescein in situ apoptosis detection kit (EMD Millipore) following the manufacturer's protocol. The stained slides were later examined under a fluorescence microscope (Nikon Eclipse 80i; Nikon Instruments, Tokyo, Japan).

### Whole-Mount Staining of Corneal NF-κB

The eyes were enucleated from euthanized mice and fixed at room temperature in 4% paraformaldehyde for 10 hours. The anterior segment was then removed with a razor blade. The corneas were mounted on a dome-shaped post and then were cut with a single-edge razor blade into four standardized parts. They were rinsed several times and blocked at room temperature. The corneas were incubated overnight at 4°C in anti-NF-κB (1:100, Invitrogen). After being rinsed, a secondary antibody was used. DAPI solution was used for nuclear counterstaining. The tissue was cover-slipped with the mounting medium and analyzed under a confocal microscope (TCS SP2; Leica, Heidelberg, Germany).

### Tregs and Corneas Co-Culture

Immediately after corneal alkali burn, eyes were enucleated from euthanized mice, then the corneas were excised. Tregs freshly isolated from secondary lymph nodes and spleens were co-cultured with corneas for the indicated time. 24-well transwell plates (Corning, Life Sciences), with 0.4 µm pore polycarbonate membrane insert, were used. 5 × 10^5^ Tregs/well and 4 corneas after alkali burns were cultured together for 24 hours with supplementation of anti-Areg (50 µM), control IgG (1 µM), or recombinant Areg (1 µM).

### Subconjunctival Injection of Molecular Reagents

The subconjunctival injection volume for mice was 10 µL at one site. The dosages for neutralizing anti-Areg and rmAreg were determined according to the median effective dose (ED_50_) provided by the supplier, and the maximal concentration was chosen. Anesthetized mice were injected with 10 µL/cornea Areg-neutralizing antibody (5 µg per mouse) or rmAreg (1 µg per mouse) using a 33-gauge metal needle and a 50-µL syringe. Isoform-matched IgG (1 µg per mouse) was served as the control.

### RNA Isolation and Real-Time PCR

Total RNA was extracted from corneas from each group using a Rneasy Mini Kit (Qiagen, Valencia, CA, USA). The concentration and purity of the total RNA were analyzed spectrophotometrically at OD_260 nm_ with OD_280 nm_ as a reference. Afterwards, quantitative PCR (qPCR) analyses were performed on high-quality RNA samples with *A*_260_/*A*_280_ ratios between 1.9 and 2.1. RNA using a PrimeScript RT reagent kit (TaKaRa, Dalian, China) was used to reverse transcribe cDNA. Real-time PCR was conducted on an ABI Prism 7000 instrument using SYBR Green PCR Master Mix (Life Technologies). Data represented mean ± SEM of three independent experiments. Each time point of one group consisted of four mice. Primer sequences are shown in the Table.

**Table.** Primers Used for Reverse Transcriptase qPCR

Genes	Forward (5′-3′)	Reverse (3′-5′)
**NF-κB**	TTTGCAACTATGTGGGGCC	GCGTGCAGGTGGATGTT
**NLRP3**	GCTGGCATCTGGGGAAACCT	CTTAGGCTTCGGTCCACACA
**Caspase-1**	GCCTGTTCCTGTGATGTGGA	TTCACTTCCTGCCCACAGAC
**Caspase-3**	GAGCTTGGAACGGTACGCTA	GAGTCCACTGACTTGCTCCC
**Caspase-8**	AAGCAGGAAGTGTGAGAGGC	GATCCTCAGGAGGCACCTTG
**IL-1β**	TGAAATGCCACCTTTTGACAG	CCACAGCCACAATGAGTGATAC
**MCP-1**	TGCCCTAAGGTCTTCAGCAC	AAGGCATCACAGTCCGAGTC
**TNF-α**	AGGCACTCCCCCAAAAGATG	CTTGGTGGTTTGCTACGACG
**iNOS**	CAAGCACCTTGGAAGAGGAG	AAGGCCAAACACAGCATACC
**CD86**	TTGTGIGTGTTCTGGAAACGGAG	AACTTAGAGGCTGTGTTGCTGGG
**CD206**	TCTTTGCCTTTCCCAGTCTCC	TGACACCCAGCGGAATTTC
**IL-10**	CTTACTGACTGGCATGAGGATCA	GCAGCTCTAGGAGCATGTGG
**CK12**	CAGACCTTGGGGTGCATCTG	CCACCGCTAAAGCCAGAACTA
**ZO-1**	GAGCAGGCTTTGGAGGAGAC	TGGGACAAAAGTCCGGGAAG
**β-Catenin**	GTCAGTGCAGGAGGCCG	GGCCATGTCCAACTCCATCA
**Pax6**	CGTAGAACCCGGTTGTCAGA	GTGTCAGGTGAGTCTGGTGG
**Bax**	CTGGATCCAAGACCAGGGTG	GTGAGGACTCCAGCCACAAA
**BCL-2**	GAACTGGGGGAGGATTGTGG	GCATGCTGGGGCCATATAGT
**CD44**	CACCTTGGCCACCACTCCTA	TTCTICCCCTGCCAICCGTT
**GITR**	CAAGCACTACCCCTGCCAAC	AGCATTGTGGGTCTTGTTCCC
**ICOS**	GGCAGACATGAAGCCGTACT	GCCGAGCCATTGATTTCTC
**CTLA-4**	GGGCTTTCCAAACTGGGT	ACATTCTGGCTCTGTTGGGG
**Ki67**	CAGAGCTAACTTGCGCTGAC	CGCTTGATGGTGACCAGGTG
**PCNA**	AAAGATGCCGTCGGGTGAAT	CCATTGCCAAGCTCTCCACT
**EGFR**	GTGGCCATCTGGGTACGTT	AAGGTGAGAGGGGAGTCAGAG
**Acta2**	GTACCACCATGTACCCAGGC	GCTGGAAGGTAGACAGCGAA
**Col3al**	GCGAGCGGCTGAGTTTTATG	GCAGCTCAGAGTAGCACCAT
**Tenascin C**	CTCCCAGCATCCGTACCAAA	CCAGGAAACTGTGAACCCGT
**VEGF A**	TATTCAGCGGACTCACCAGC	AACCAACCTCCTCAAACCGT
**GAPDH**	AGGAGAGTGTTTCCTCGICC	TGCCGTGAGTGGAGICATAC

### Enzyme-Linked Immunosorbent Assay (ELISA)

The cell-free supernatants were collected from cell cultures after centrifugation at 1500 rpm for 4 minutes at 4°C and assayed for targeted proteins. The cultured cells were digested and centrifuged at 800 rpm for 4 minutes at 4°C to collect the cell precipitate. Afterwards, the cells were lysed using RIPA Lysis and Extraction Buffer (Thermo Fisher Scientific) in the presence of protease inhibitors (Roche) and then were sent for detection of protein levels of specific cytokines by enzyme-linked immunosorbent assay (ELISA) kits. Four corneas from the same group were extracted and homogenized in 0.01 M sodium phosphate buffer (pH 9.5) containing protease/phosphatase inhibitor cocktail (1:100, Sigma) on ice. Commercially available ELISA kits were used following the manufacturer's protocols to measure the protein levels of NLRP3, IL-1β, IL-10, Areg, TNF-α, TGF-β, and VEGF-A (ELK Biotechnology). Each sample was assayed in three technical replicates. Data represented mean ± SEM of three independent experiments, and each time point of one group consisted of four mice.

### Statistical Analysis

Data represented mean ± standard error of mean (SEM). All experiments were performed with at least three repeats. Data were analyzed using one-way analysis of variance followed by Tukey's multiple-comparison test to compare the means of more than two groups and two-tailed Student *t*-test was used to compare parameters between the two groups. Statistical analysis and graphical representation of data were done using the software of GraphPad Prism (La Jolla, CA, USA). A significant difference in means was indicated as follows: ^*^P < 0.05, ^**^P < 0.01, ^***^P < 0.001, and ^****^P < 0.0001.

## Results

### Accumulation of Tregs on the Ocular Surface After the Corneal Alkali Burn

**Figure 1.**
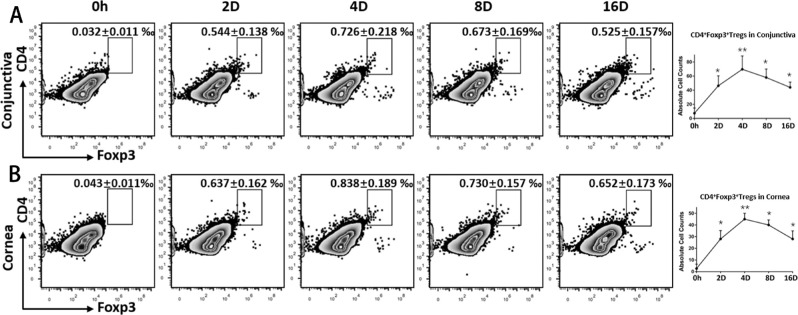
**Kinetic distribution of Tregs on the ocular surface after corneal alkali burn.** Excised corneas and conjunctiva were harvested and made into single-cell suspension to analyze the distribution of Tregs at different time points after the corneal alkali burn. Zero-hour (0-h) data were derived from mice not injured by alkali. Representative flow cytometric dot plots show the frequencies of CD4^+^Foxp3^+^ Tregs on the ocular surface (conjunctiva (**A**) and cornea (**B**)) at different time points after the corneal alkali burn. The right panels show absolute Treg count in different tissues.

After an acute corneal sterile injury, inflammatory cells exaggerated damages by releasing the influx of various deleterious cytokines and leukocytes.^15^ As maintainers of immunological homeostasis, Tregs have been reported to recruit to corneas in virus-infected keratitis models.^16^ Corroborating the findings of fluorescent activated cell sorting (FACS) analyses, Tregs infiltrated the local site of acute corneal alkali burn and continuously accumulated (Fig. 1), which suggested their intrinsic protective role in acute corneal damage.

### Subconjunctivally Injected Tregs Promoted Corneal Healing by Improving Re-epithelialization and Alleviating Corneal Edema

**Figure 2.**
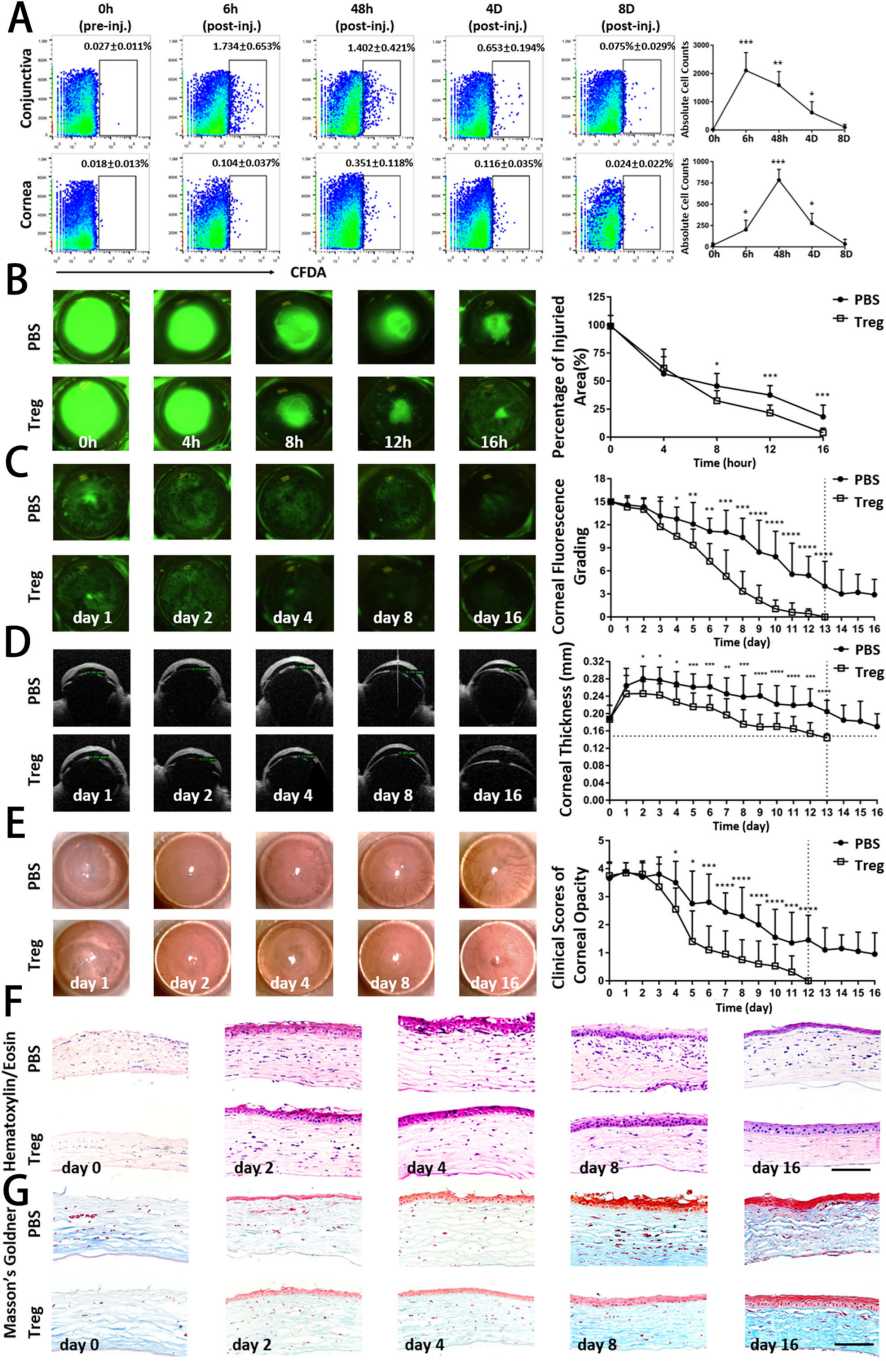
**Clinical examinations and histological analysis revealed an improved corneal restoration by treatment with subconjunctival injection of Tregs.** (**A**) The CD4^+^CD25^+^ Treg cells were isolated and labeled with CFDA-SE. The 1 × 10^5^ Tregs/10 µL of PBS were injected subconjunctivally immediately after the corneal alkali burn. The control group was treated with 10 µL of PBS. The injured cornea and ipsilateral conjunctiva were digested and analyzed for labeled Tregs using flow cytometry. Representative flow cytometry dot plots show frequencies of Treg cells stained with CFDA at different time points after injection (pre-inj., pre-injection; post-inj, post-injection). (**B**) A 2-mm wound was made in the center of the cornea, and the healing process was visualized with fluorescein staining to highlight the denuded area. Representative images during the acute stage of corneal alkali burn injury (0–16 h) are shown. The percentage of corneal epithelial defective area in both groups at each time point was analyzed and summarized in the right panel. (**C**) Representative ocular surface fluorescein staining images of mice with corneal alkali burn during the latter recovery and reconstruction stage of injury (days 1–16) are shown. The corneal epitheliopathy evaluated by the National Eye Institute score in both groups at each time point was analyzed and summarized in the right panel. (**D**) Representative optical coherence tomography (OCT) images showed the corneal thickness at various time points. The horizontal dotted line shows the thickness of the corneas of normal mice. Corneal thickness measured by OCT are summarized in the right panel. (**E**) Representative bright-field corneal images showed corneal opacity at various time points. Clinical scores of corneal transparency are summarized in the right panel. (**F**) Representative hematoxylin and eosin staining images of alkali-injured corneas at serial time points in Treg-treated and PBS-treated groups are shown. Scar bar: 50 µm. (**G**) Representative Masson's Goldner (MG) staining images of normal and alkali-injured corneas are shown. Blue staining in the stroma represents collagen fibers, and red staining represents muscle fibers. Scar bar: 50 µm. Data shown represented three independent experiments. Statistical comparisons were made between the Tregs-treated and the PBS-treated groups at each individual time points.

Next, the gain-of-function experiment was performed to distinguish the function of Tregs in the development of corneal injury after the alkali burn. CD4^+^CD25^+^ Tregs were isolated and injected subconjunctivally immediately after the corneal alkali burn. Tregs were labeled with CFDA-SE to track the kinetic distribution of Tregs on the ocular surface. In Figure 2A, Tregs were predominantly detected as early as within 6 hours in injured corneas. They reached the maximal number in 48 hours, followed by a gradual decrease, and then became nearly undetectable after 8 days.

Alkali reagents impair the corneal epithelium and penetrate the stroma.^17^ The ocular surface was stained with fluorescein and examined using a slit-lamp microscope so as to assess the effect of Tregs on corneal epithelial wound healing. As shown in Figure 2B, a green disk-shaped burned area was observed in the center of the corneas. The corneal wound closure did not show much difference 4 hours after wounding between Treg-treated and control groups. However, 8 hours later, the closure of Treg-injected wounds was accelerated, as illustrated by smaller wound areas compared with the control group. In 16 hours, almost all burned areas were covered by the epithelium in the Treg-treated group, while there was still about 25% of the area that had epithelial defects in the control group, demonstrating a potential role of Tregs in promoting re-epithelialization. The corneal epitheliopathy NEI scores (Fig. 2C) were determined to evaluate the subsequent corneal re-epithelization process. The scores decreased from 15 to 0 on day 13 in the Treg-treated group, indicating the complete abolishment of epithelial defects. In contrast, the control group showed a much slower decrease in scores and continued suffering from epithelial keratopathy on day 16 (with an average score of 3). Corneal edema was evaluated by OCT to measure the thickness (Fig. 2D). The differences between the two groups progressively increased over time, with a much faster reduction of corneal thickness in the Treg-treated group. The opacity scores in the two groups were also evaluated (Fig. 2E). The consistently changing patterns exhibited that the Treg treatment effectively improved corneal transparency. At the end of the observation, complete recovery from alkali injury was noted in the Treg-treated group, while the control group still exhibited a moderate lack of opacity.

The histological analysis was conducted to examine detailed structural repair differences between the two groups. As shown in Figures 2F, 2G, the laminated squamous epithelium was completely exfoliated, and moderate edema with disordered collagen fibers was found in the stromal layer after alkali injury. A pathological corneal morphology was still observed on day 16. Notably, a relatively intact and well-defined epithelium with some keratinized corneal epithelial cells having mere pathological signs was found in the Treg-treated group, which was almost indistinguishable from naïve, unwounded corneas.

Altogether, these findings indicated that the adoptive transfer of Tregs effectively exhibited their reparative properties in counteracting the development of corneal alkali injury.

### Tregs Effectively Attenuated Excessive Inflammatory Response and Promoted Macrophage Polarization in Injured Corneas

**Figure 3.**
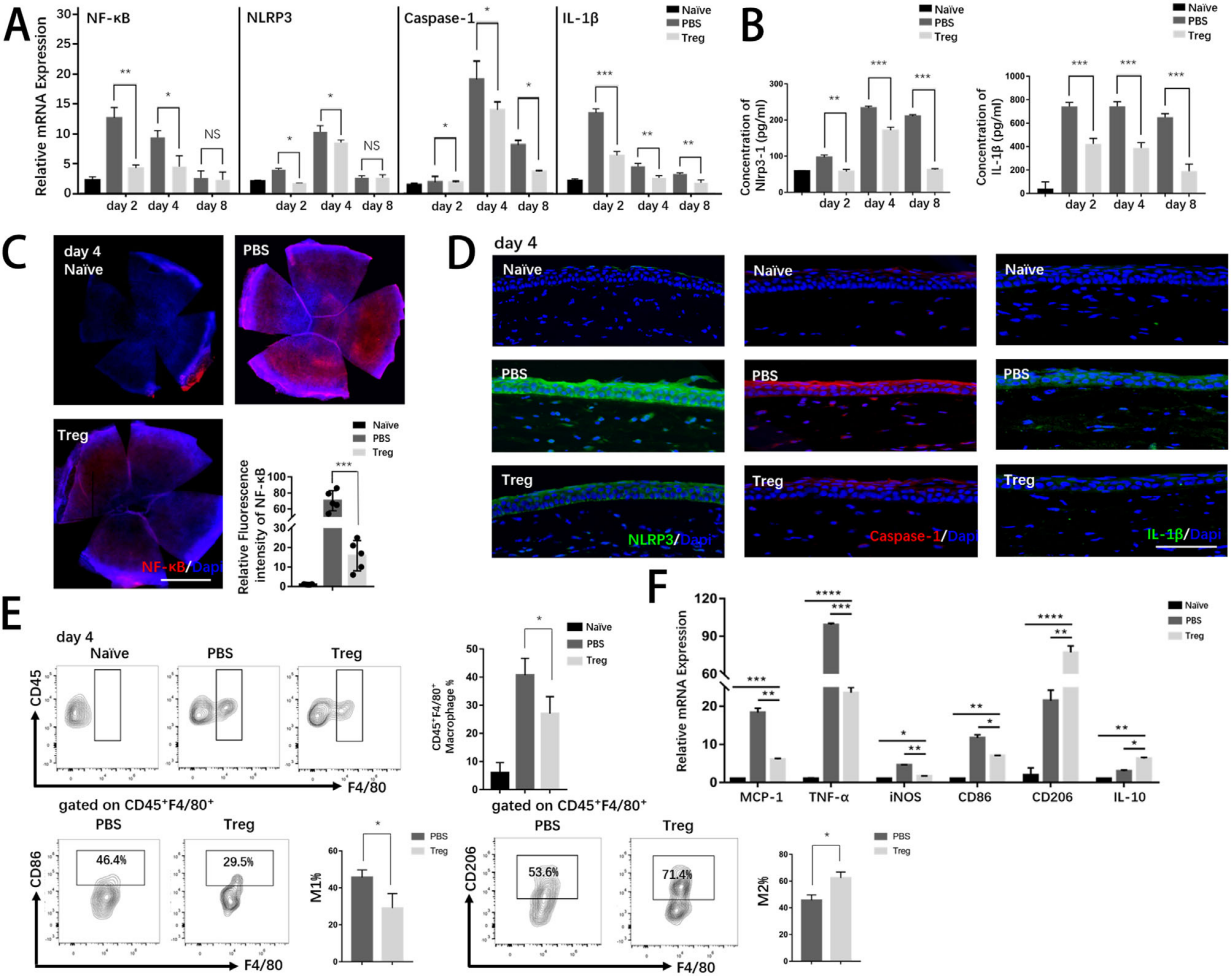
**Treg treatment suppressed excessive inflammation by inhibiting the activation inflammatory pathway and promoting macrophage polarization.** (**A**) The mRNA levels of pro-inflammatory cytokines (NF-κB, NLRP3, caspase-1, and IL-1β) were evaluated by PCR. (**B**) Protein levels of NLRP3 and IL-1β were analyzed by ELISA. (**C**) Immunostaining of NF-κB was conducted in whole corneal tissue. Nuclei were counterstained with DAPI. Scar bar: 1 mm. The relative fluorescence intensity of NF-κB analyzed by ImageJ is shown. Each group consisted of five mice. (**D**) Representative merged immunofluorescence staining images of inflammatory markers (NLRP3, caspase-1, and IL-1β) are presented. Nuclei were counterstained with DAPI. Scar bar: 50 µm. (**E**) Representative flow cytometric contour plots exhibited infiltration of corneal CD45^+^ F4/80^+^ macrophages in naïve and alkali-injured mice on day 4. Representative flow cytometric contour plots showed frequencies of CD45^+^F4/80^+^CD86^+^ (M1) macrophages and CD45^+^F4/80^+^CD206^+^ (M2) macrophages.

Corneal alkali burn is severe, contributing to overwhelming sterile inflammatory responses. Studies have shown that the innate immunity plays a vital role in the pathologic process.^18^ Particularly, macrophages, central roles in innate immunity,^19^ lead to development of pro-inflammation and neovascularization, which are major complications of alkali-injured corneas. Thus, we further examined whether the injection of Tregs had impact on innate immune responses after the corneal alkali burn. The transcription factor-nuclear factor (NF-κB) is an important signaling pathway mediating inflammatory response,^20^ and nucleotide-binding oligomerization domain-like receptor family pyrin domain-containing 3 (NLRP3) is vital in activating proinflammatory pathways.^21^ Therefore, the expression levels of associated proinflammatory cytokines in corneas were examined (Figs. 3A–D). The results showed that alkali burns led to the significantly activated proinflammatory signaling pathway (NLRP3-Caspase-1-IL-1β) followed by a cascade of downstream inflammatory events. With effective treatment using Tregs, inflammation was inhibited at both mRNA and protein levels.

Macrophages are implicated in excessive inflammation in response to tissue damage and closely correlated with corneal neovascularization. Therefore, we investigated the effect of anti-inflammatory properties of Tregs on macrophage response using FACS (Fig. 3E). The ratio of macrophages (M1, inflammatory phenotype, characterized by CD45^+^F4/80^+^CD86^+^) decreased in corneas, while M2 (anti-inflammatory phenotype, characterized by CD45^+^F4/80^+^CD86^+^) increased after Treg-treatment, which suggested that Tregs could promote M2 polarization and therefore limit the expression of proinflammatory molecules. The effects of Treg on other immune cells, such as mast cells and neutrophils will be examined in future investigations.

To sum up, Treg treatment alleviated the inflammatory response, accounting for improved clinical findings in the aforementioned results.

### Tregs Promoted the Proliferation of Epithelial Cells and Inhibited Corneal Apoptosis

**Figure 4.**
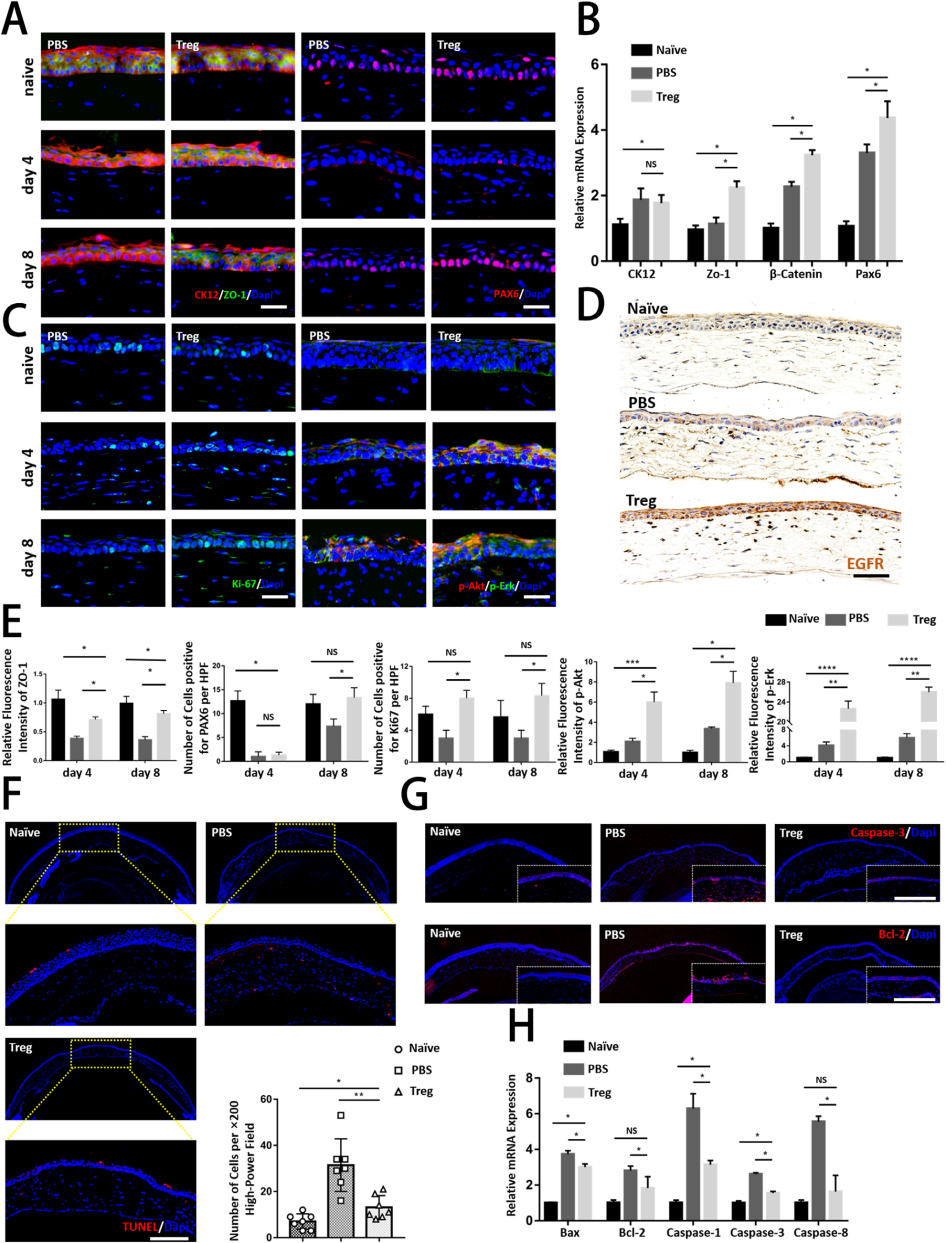
**Tregs recovered cornea structurally and functionally by promoting proliferation and inhibiting the apoptosis of corneal epithelial cells.** (**A**) Representative immunofluorescence micrographs of corneas stained with CK12 (a corneal epithelial marker), ZO-1 (an epithelial tight junction marker), and PAX6 on days 4 and 8 after the injury. Scar bar: 25 µm. (**B**) Relative mRNA expression of corneal structural and functional markers (CK12, ZO-1, β-Catenin, and Pax6) is shown. (**C**) The regenerating corneal epithelium was stained with Ki-67 (a proliferation marker), p-Akt, and p-Erk on days 4 and 8 after the injury. Scar bar: 25 µm. (**D**) Representative immunofluorescence micrographs of corneas stained with EGFR are shown. Scar bar: 50 µm. (**E**) The panels analyzed the relative fluorescence intensity and number of cells per high-power field (HPF) in above immunofluorescence graphs. (**F**) TUNEL staining was conducted in corneas. Staining of seven corneas in each group was quantified by calculating the percentage of TUNEL^+^ cells in each image. Scar bar: 50 µm. (**G**) Representative immunofluorescence micrographs of corneas stained with caspase-3 and Bcl-2 are shown. Scar bar: 50 µm. (**H**) Relative mRNA expression of apoptosis genes (Bax, Bcl-2, caspase-1, caspase-3, and caspase-8) is shown.

Serial histological analysis was performed to further evaluate the healing process after treatment with Tregs. Double staining was performed for CK12, a corneal epithelium marker,^22^ and ZO-1, a marker of tight junctions.^23^ As shown in Figure 4A, the Treg-treated group showed intense staining of ZO-1, with a quite similar staining pattern as normal corneas, while the control group showed less and weaker staining, indicating that Tregs might help maintain corneal integrity. Staining of PAX6, a marker representing the maintenance of the proper functioning of corneal epithelial cells,^24^ was also performed to analyze functional differences after Treg treatment. The results showed a higher number of positive cells in the basal layers of corneas on day 8 in the Treg-injected group compared with the control group, indicating that Treg treatment might specifically activate epithelial cells to repair corneal injury. Consistently, the mRNA levels of corneal structural and functional markers were also significantly upregulated by Tregs (Fig. 4B).

Cell proliferation was evaluated by analyzing the nuclear staining of the proliferation marker Ki67 (Fig. 4C). The results showed that the differences between the two groups were highly significant for the epithelium, with a higher number of positive cells on days 4 and 8 in the Treg-injected group. The activated proliferative pathway contributing to more positive Ki67^+^ cells after Treg treatment was further examined. As the intracellular PI3K-kinase/Akt and MAPK/ERK cascades are generally two of the most important signaling pathways related to cell proliferation,^25^ the Akt and ERK phosphorylation levels were measured, which were noticeable upregulated after injury and meanwhile Treg-treated corneas exhibited more intense staining. Expectantly, higher expression levels of the epidermal growth factor receptor (EGFR), which was responsible for the activation of phosphorylation of the two pathways (Fig. 4D), were detected after Treg treatment. The relative fluorescence intensity of the mentioned markers was analyzed in Figure 4E.

Considering the critical role of apoptosis in triggering corneal dysfunction and determining the early pathophysiology of corneal alkali burns,^26^ the study next explored the effect of Tregs on corneal apoptosis in the epithelium and stroma (Fig. 4F). The data showed that Tregs significantly protected corneas from the alkali challenge, as evidenced by TUNEL^+^ apoptotic cells and suppression of important factors during apoptosis at mRNA and protein levels in Treg-treated corneas (Figs. 4G, 4H).

To conclude, Tregs could repair the wounded corneas morphologically and functionally with the stabilization of the epithelial barrier, activation of corneal epithelial cell proliferation, and inhibition of apoptosis.

### Local Treg Injection Upregulated the Expression of Corneal Areg

A Treg–cornea co-culture protocol was performed in vitro, and the phenotypic and functional statuses of Tregs stimulated by alkali-burned corneas were evaluated to determine the intrinsic mechanism by which recruited Tregs counteracted the development of inflammation after corneal injury.

**Figure 5.**
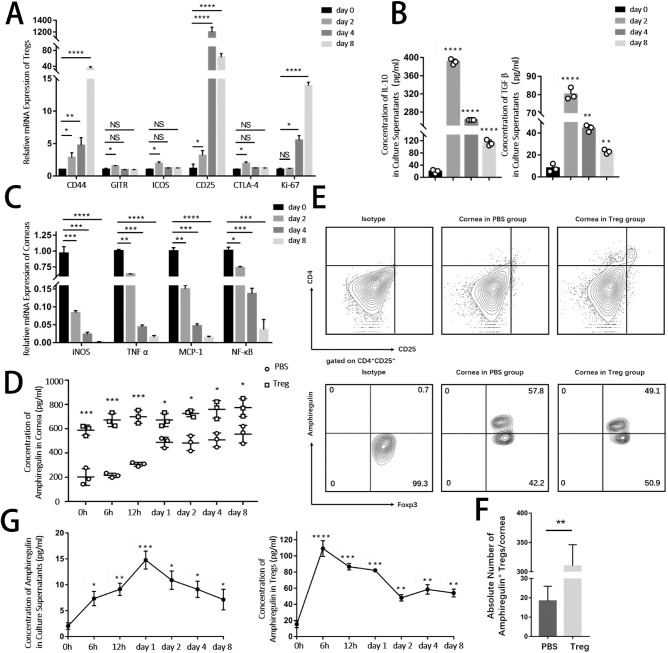
**Tregs had higher levels of functional markers, including amphiregulin after being stimulated by alkali-injured corneas.** Freshly isolated Tregs were co-cultured with alkali-burned corneas using the Transwell System. (**A**) Expression of markers related to Tregs (CD4^+^CD25^+^Foxp3^+^) function and activation (CD44, GITR, ICOS, CD25, CTLA-4, and Ki67) after co-culturing with damaged corneas for 2, 4, and 8 days in vitro was evaluated. (**B**) The protein levels of anti-inflammatory cytokines IL-10 and TGF-β in culture supernatants were analyzed by ELISA. (**C**) Relative mRNA expression of pro-inflammatory cytokines in corneas in vitro cocultured with Tregs was examined. (**D**) Representative flow cytometry plots showing the amphiregulin expression in corneas from PBS and Treg groups (gated on live cells). (**E**) The lower panel analyzed the number of Areg^+^ Tregs per cornea. (**F**) Kinetics protein levels of amphiregulin in alkali-injured corneas at different time points after Tregs or PBS (control) treatment. (**G**) Concentration of amphiregulin protein in supernatants and in Tregs was examined by ELISA.

The most significantly upregulated molecules found in Tregs are CD44, GITR, ICOS, CD25, CTLA-4, and Ki67, which are markers of activation and functional suppression of Tregs (Fig. 5A). Corroborating these data, an increase in the levels of anti-inflammatory cytokines, IL-10 and TGF-β, was found in culture supernatants in Treg–cornea co-culture system along with a dramatic decrease in inflammatory responses in dissected corneas after co-culturing with Tregs (Fig. 5B).

Tregs can participate in the repair of damaged tissues. Therefore, the present study explored the participation of Areg and keratinocyte growth factor (KGF), which have been reported to result in the activation of downstream signaling kinases inducing the growth, proliferation, and migration of cells in various tissues.^27^ The concentration of Areg in corneas were much higher in the Treg group than the PBS group (Fig. 5D), while there were no significant differences of KGF between two groups (data not shown), indicating that Tregs could upregulated Areg to exert their protective function. The speculation was further confirmed flow cytometry. More than 48% of Tregs that migrated to corneas exhibited positive expression of Areg (Fig. 5E). It was deduced that the microenvironment of sterile injured corneas could stimulate Tregs to secrete abundant Areg to take part in tissue regeneration. An emerging body of literature suggests that tissue-resident Tregs have specialized functions that are unique to the tissues in which they reside. We speculate that it was the reason of a comparatively high expression of amphiregulin in autologous Tregs. Despite that the expression levels of amphiregulin in injected Tregs were not as high as autologous corneal Tregs, the number of Areg^+^ Tregs in corneas was significantly higher in Tregs group, which therefore implied that subconjunctival injection of Tregs could secrete abundant amphiregulin. In in vitro experiment (Fig. 5G), after co-culturing with damaged corneas for 6 hours, the concentration of Areg increased rapidly in Tregs, followed by a peak in culture supernatants on day 1. The high concentration of Areg in culture supernatants was in line with faster corneal re-epithelialization, thus suggesting that the Areg production of Tregs was involved in corneal healing.

Taken together, the data suggested that newly recruited Tregs on the ocular surface were activated upon stimulation by inflammatory corneas to express functional markers, especially Areg, to exert their reparative capacity on corneas.

### Effect of Treg Treatment on the Alkali Burn Depended on the Upregulation of Areg

**Figure 6.**
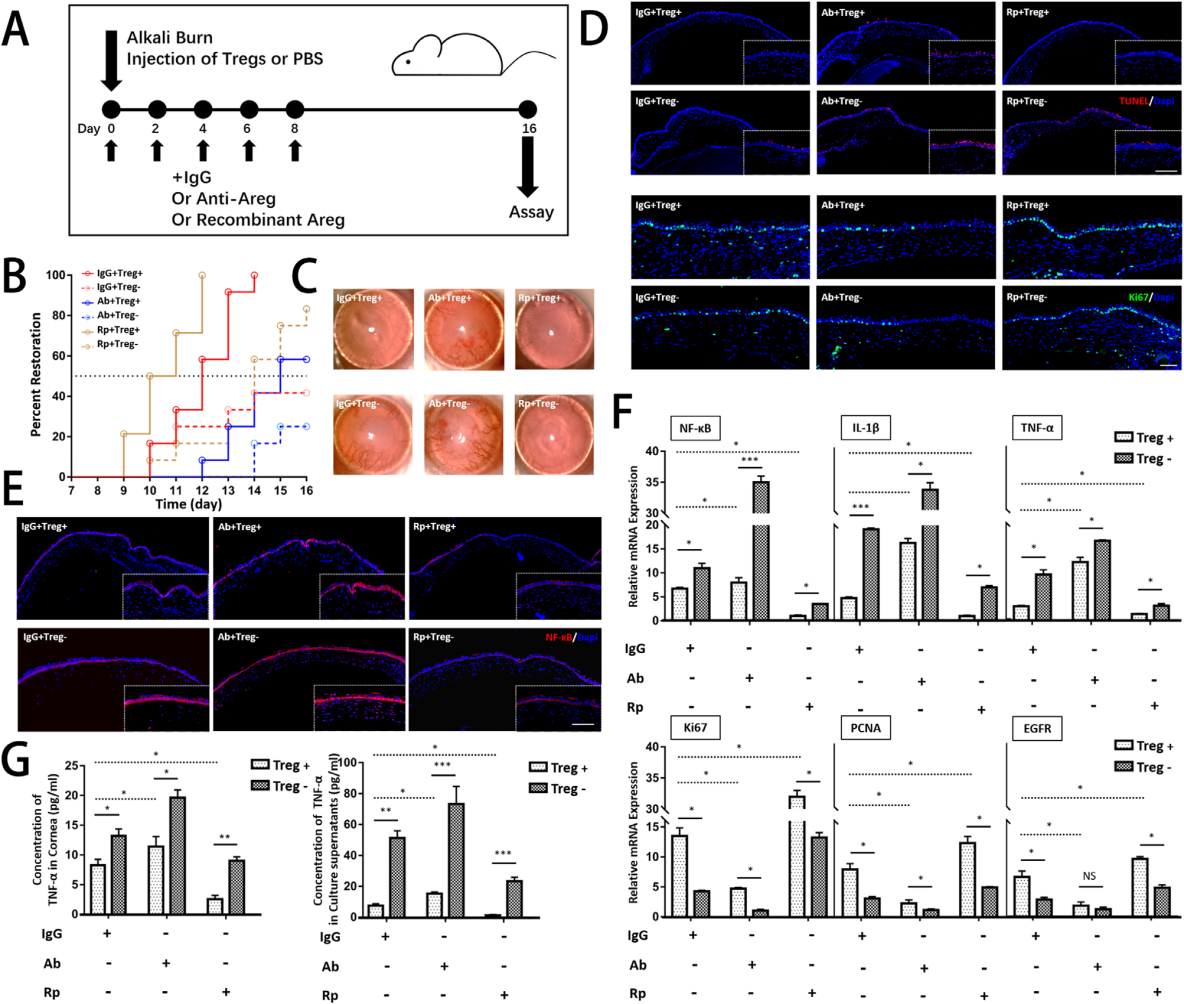
**Tregs promoted corneal healing through an amphiregulin-dependent mechanism.** (**A**) Schematic diagram of the study time points and specific treatments is shown. (**B**) The corneal restoration rate was analyzed by evaluating the scores of NEI and OCT. Complete abolishment of fluorescence staining in corneas and recovery of corneal thickness measured by OCT (less than 0.142 mm) were determined as complete corneal restoration. (**C**) Representative bright-field corneal images of six groups showed corneal transparency on day 16. (**D** and **E**) Immunostaining of TUNEL, Ki67, and NF-κB is presented. Scar bar: 100 µm. (**F**) The mRNA levels of inflammatory cytokines and proliferative cytokines in corneas were analyzed in corneas by PCR. (**G**) ELISA was conducted to examine the protein levels of TNF-α in corneas and supernatants in Tregs–cornea co-culture system.

Based on previous findings, this study next determined whether an Areg-dependent mechanism was responsible for the modulatory effect of Tregs on the corneal repair. Alkali-injured mice were treated with rmAreg (Rp^+^), anti-Areg (Ab^+^) and IgG with or without Tregs after alkali injury. A schematic diagram of the study time points and specific treatments is shown in Figure 6A.

The corneas healed the fastest using a combination of Tregs and rmAregs, with complete recovery in corneal opacity and edema on day 12 (Fig. 6B), whereas anti-Areg-treated corneas without Tregs exhibited the worst restoration. After blunting the function of Areg, Treg treatment was much worse than IgG^+^Tregs^+^ treatment, suggesting the participation of Areg in corneal repair. The gain-of-function experiment further verified the hypothesis, indicating that rmAreg treatment significantly improved corneal recovery compared with single-IgG treatment. Representative slit-lamp photographs are shown on the right in the figure, presenting differences in corneal opacity after different treatments, which were consistent with the results of corneal restoration.

TUNEL staining (Fig. 6D) showed corneal apoptosis after different treatments. Single treatment of rmAregs as well as Tregs could reduce apoptosis in corneas with less positive staining of TUNEL^+^ cells. Corneas treated with a combination of rmAreg and Tregs exhibited the least positive TUNEL staining. Anti-Areg increased the staining of TUNEL^+^ cells in the Treg-treatment group. Without Treg injection, the anti-Areg group exhibited the strongest staining of apoptotic cells, probably due to the neutralization of intrinsic Areg. The mRNA levels of apoptotic cytokines were determined, and the results were in line with immunostaining results.

Based on previous results that a Treg injection could stimulate corneal epithelial cells by increasing Ki67 expression, it was hypothesized that Treg-induced Areg might be a critical factor involved in regeneration. Exogenous rmAreg augmented cell proliferation with stronger staining of Ki67 compared with corneas treated with IgG, while the proliferative effects were abrogated by anti-Areg administration, presenting corneas with weaker Ki67^+^ compared with those receiving IgG treatment, probably due to the blunting of intrinsic Areg by the antibody. The results were indicative of the involvement of Areg in corneal proliferation. The proliferation of corneal epithelial cells was suppressed after injecting the combination of anti-Areg and Tregs, with the weaker staining of Ki67 compared with that in cells with no anti-Areg treatment. The results of PCR examining the levels of regenerative markers were in accordance with immunostaining findings, demonstrating that the regenerative function of Tregs partly diminished after blocking Areg, indicating an Areg-dependent functioning of Tregs.

Consistently, the results of ELISA conducted on the Treg–cornea co-culture system to analyze the direct effects of Areg also confirmed that rmAreg could induce the suppression of TNF-α in both corneas and supernatants. The combination of anti-Areg and Tregs reverted the effects of alkali-primed Tregs by showing no significant changes, suggesting that Areg imparted Tregs the lesion resolution potential in alleviating inflammation and promoting cell proliferation; the results agreed with other reports on the tissue protection of Tregs via the upregulation of Areg during corneal stromal keratitis.^28^

To sum up, Tregs could impede cell apoptosis and promote proliferation of corneal epithelial cells via the expression of Areg.

### Prompt Transfer of Tregs in the Acute Stage Led to Improved Prognosis by Preventing Corneal Fibrosis and Neovascularization

**Figure 7.**
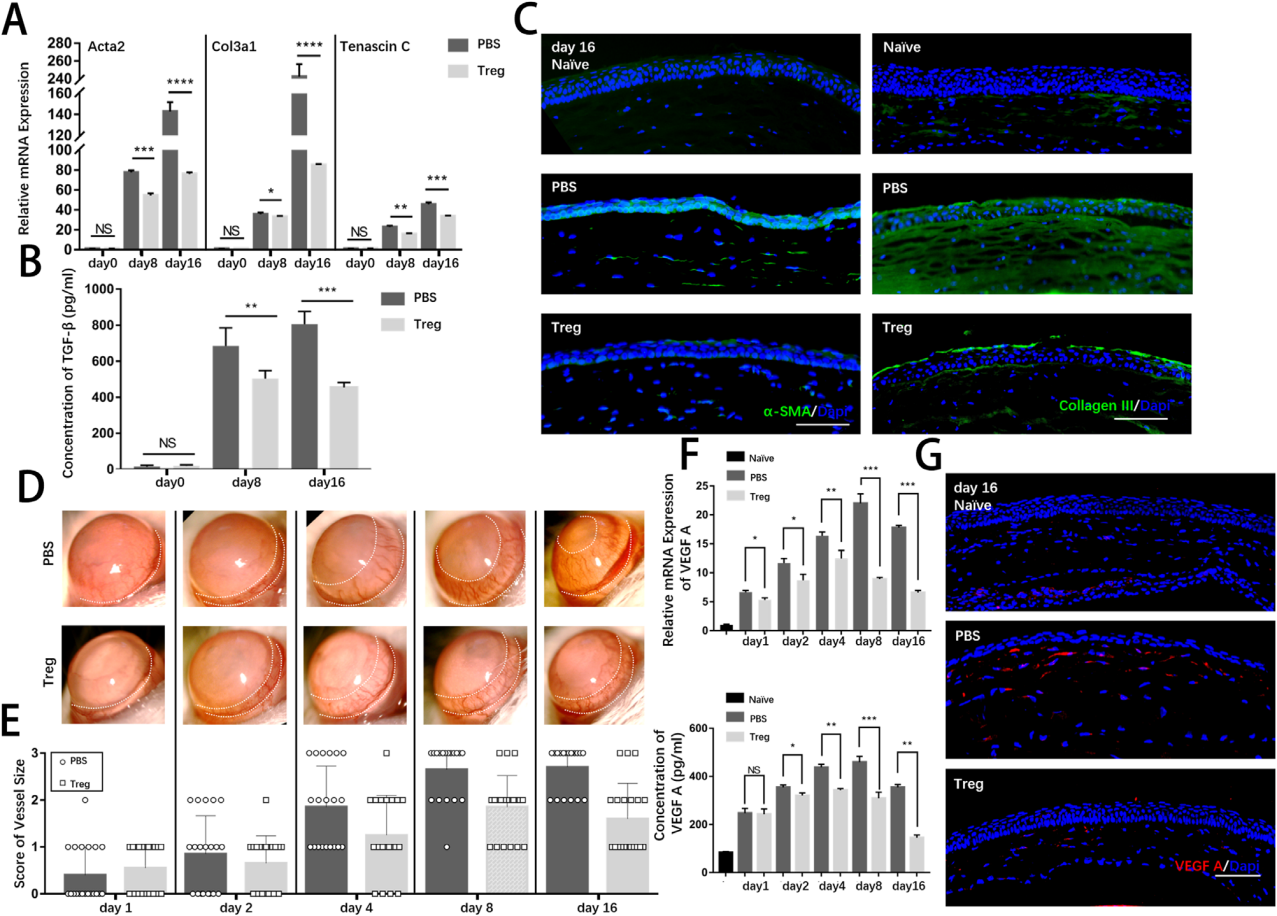
**Treg injection improved prognosis of corneal alkali burns with reduced corneal fibrosis and decreased corneal neovascularization during the tissue remodeling stage.** (**A**) The relative mRNA expression of Acta2, Col3a1, and tenascin C in corneas is shown. (**B**) ELISA examined the protein levels of TGF-β. (**C**) Representative immunofluorescence micrographs of corneal stroma stained with anti-α-SMA and collagen III on days 8 and 16 are presented. Scar bar: 100 µm. (**D**) Representative bright-field corneal images showed the development of neovascularization in the corneal limbal area at various time points. (**E**) Clinical scores of corneal neovascularization are summarized in the right panel. (**F**) Both mRNA and protein levels of VEGF A are shown. (**G**) Representative immunofluorescence micrographs of cornea stained with VEGF A on day 16 are presented. Scar bar: 100 µm.

Excessive inflammatory responses are closely related to corneal fibrosis and neovascularization, two major complications after corneal alkali burns that contribute to diminished visual acuity or even permanent blindness.^29^ In previous studies using Masson trichrome staining (Fig. 2G), corneal fiber structures were visualized after the alkali injury. Intriguingly, the differences between corneas with PBS injection and corneas with Treg injection on day 16 were obvious. The control group exhibited disordered muscle fibers with a larger extent of collagen deposition in the stroma, indicating the formation of corneal scar and low transparency. The orientation of collagen fibers was much more organized in the Treg-treated group, with much resemblance to naïve corneas. PCR was conducted to analyze the expression of myofibroblast marker smooth muscle actin (Acta2) and fibrotic marker collagen 3 (Col3a1) at the mRNA level to further investigate the changes at molecular levels. As shown in Figure 7A, the expression of pro-fibrotic markers was markedly upregulated after wounding. Consistently, Tregs suppressed the mRNA levels of fibrotic markers at the beginning of tissue remodeling. The protein levels of TGF-β were examined (Fig. 7B), which is a major factor responsible for tissue fibrosis and scar formation.^30^ The results showed a significant reduction in corneas after Treg treatment. The expression of α-smooth muscle actin (α-SMA), a protein produced by myofibroblasts, and collagen III, deposition of excessive extracellular matrix, were examined by immunofluorescence (Fig. 7C). The corneas in the control group exhibited dramatically and progressively increased expression of α-SMA and collagen III on days 8 and 16 after the alkali burn, while the fibrotic formation was abrogated in those in the Treg treatment group, which was in line with the clinical opacity score data (see Fig. 2E).

Corneal neovascularization is another major complication leading to poor prognosis.^3^ Therefore, this study evaluated the kinetic growth of corneal neovascularization (Fig. 7D). The vessel size was scored under the bright-field slit lamp throughout the tissue remodeling stage (Fig. 7E), revealing that corneal neovascularization steadily progressed after the alkali burn but Treg treatment significantly reduced the adverse outcome. The upregulation of VEGF-A, a valuable pro-angiogenic indicator of angiogenesis after corneal alkali injury, was significantly suppressed by Tregs (Fig. 7F). A similar change in the protein levels of VEGF-A was observed in the two groups, evidenced by strong staining signals in the control corneas and much weaker staining in the Treg-treated corneas (Fig. 7G).

## Discussion

Corneal alkali burn is a major health burden disrupting the overall functioning and quality of life, with continuous influx of recruited leukocytes and inflammatory cytokines.^31^ As major endogenous counter-regulatory mechanisms of inflammatory responses and therapeutic efficacy in treating various tissue injuries,^32^^–^^34^ Tregs-treatment has also been studied in facilitating wound repair for eye diseases.^35^ In our study, it was the first time to investigate the therapeutic effects of Tregs in alkali-injured corneas. Injected Tregs were enriched in inflamed corneas. Faster re-epithelialization, quicker alleviation of corneal edema, significant improvement in corneal opacity, and better structural and functional recovery were observed in the Treg-treated group compared with the control group. The results demonstrated that the adoptive transfer of Tregs could be an enticing prospect at the ocular surface.

The distinct immunoregulatory capacity of Tregs might be partly responsible for their effectiveness in treating corneal alkali burns since excessive inflammation is closely associated with sight-threatening corneal ulceration and perforation. We found that Tregs could promote polarization of macrophages, which are recognized as a critical part in excessive corneal inflammation in response to tissue damage and closely correlated with corneal neovascularization. Cytolysis, modulation of dendritic cell function, secretion of anti-inflammatory factors, and metabolic competition are important mechanisms of action of Tregs to exert their regulatory function in inflamed tissues.^36^ Anti-inflammatory cytokines IL-10 and TGF-β have been reported to play a potent and indispensable role in modulating mucosal immune responses.^37^^,^^38^ Consistently, our data show upregulation of both cytokine levels in the supernatants of Treg – alkali-injured cornea co-cultures, suggesting that increase of anti-inflammatory cytokines could be one of mechanisms by which the injected Tregs exert immunoregulatory function in corneal alkali burn. The upregulation of immune regulatory functional markers of Tregs – CD25 and CTLA-4 in Treg-treated group also supported our hypothesis. Upregulation of CD25 has been reported to result in cytokine deprivation-mediated apoptosis of effector T cells,^39^ and CTLA-4 has been reported to modulate the function of dendritic cells (DC).^40^^,^^41^ The regulatory effect of Tregs on other immune cells will be studied in the future.

The regulatory effect of Tregs on the inflammatory microenvironment was probably responsible, at least in part, for the improvement in corneal repair, which was consistent with the finding of Alejandro that effective control of inflammation could indirectly promote corneal wound healing,^42^ their pro-regenerative capacity cannot be ignored.

The instrumental regenerative role of Tregs through the paracrine function of Areg and KGF to directly promote tissue restoration has been proved, besides the well-established role of Tregs as the regulators of an immune response.^43^^,^^44^ The FACS analysis and ELISA examination revealed a prominent upregulation of Areg on Tregs, the time-dependent increase of which was in line with the alleviation of corneal injury, suggesting that corneal injury-stimulated Tregs could upregulate Areg, thus participating in corneal healing.

Areg has displayed remarkable therapeutic effects in a number of disease models in response to signals from injured tissues.^45^ Areg gene-deficient mice have been reported to display rare homeostatic conditions, along with an impaired resolution of inflammatory challenges.^46^ Several reports demonstrated a direct promotion of tissue repair and integrity by Areg because Areg directly acted on EGFR of tissue cells to activate innate proliferative pathways.^47^ After administrating rmAreg, corneal recovery was enhanced with an increase in corneal restoration and cells staining positive for Ki67, while the efficacy of Treg treatment was partly suppressed after using anti-Areg, suggesting that Areg might be necessary for the beneficial function of Tregs in acute corneal restoration. Combined with other reports on the association between Tregs and Areg,^46^^,^^48^ it was speculated that Tregs exerted a protective role in corneal epithelial proliferation via Areg. Additionally, the supplementation of Areg downregulated the inflammation with decreased expression levels of inflammatory cytokines in corneas, while anti-Areg upregulated the inflammation; this was consistent with the reports on Areg modulating immune responses.^49^ The loss-of-function experiment using anti-Areg treatment together with Tregs presented a blunted anti-inflammatory property of Tregs. Hence, the indispensable participation of Areg in regulating inflammatory responses was suggested. To sum up, the results provided a powerful insight into the therapeutic function of Tregs via upregulating Areg in acute wound healing.

Successful wound repair requires efficient resolution of aggravated inflammation after injury because the dysregulation of alkali-elicited immune responses have the risk of inducing collateral tissue damage.^50^^–^^52^ After Treg treatment, the prognosis of corneal alkali burns improved with an apparent reduction of fibrosis and improvement in corneal transparency. It was speculated that reduction of corneal scar and neovascularization may be attributed to the efficient resolution of local inflammation by Tregs. The correlation between Tregs and tissue fibrosis has been controversial due to the secretion of TGF-β by Tregs, which is a pro-fibrotic cytokine apart from its immune-suppressive function.^53^ The analysis of TGF-β at protein levels showed that Treg treatment did not induce higher upregulation on days 8 and 16 compared with the control group. This might be caused by the decreased number of Tregs recruited to corneas on day 8 and lower levels of endogenous TGF-β from inflammatory cells benefiting from suppressed inflammation by Tregs. Injected Tregs did not serve as a major source of TGF-β in the later stage, thus avoiding the adverse effect of abundant TGF-β in promoting tissue fibrosis. Additionally, as reported in other experiments, Tregs could function as “TGF-β sinks,” sequestering TGF-β itself to maintain their anti-inflammatory capacity and preventing free TGF-β from activating fibroblasts. Overall, the temporal tuning of TGF-β expression after Treg injection went along well with the balance between its anti-inflammatory and anti-fibrotic capacity during the corneal wound healing process. Similar anti-fibrotic property of Tregs was reported in preventing bleomycin-induced pulmonary fibrosis, myocardial fibrosis, and skin fibrosis.^54^^,^^55^

As elucidated in several studies, a cross-talk between angiogenesis and inflammation is apparent. Abnormal blood vessel growth can exacerbate abnormal corneal reconstruction and result in a substantial reduction of corneal transparency. Current treatments to decrease corneal neovascularization have variable efficacy, accompanied with side effects.^56^ Notably, in the present study, Treg treatment significantly suppressed neovascularization after the alkali burn. Previous studies showed the important roles of macrophages and proinflammatory cytokines in inducing pathological neovascularization.^57^^,^^58^ It was deduced that the capacity of the anti-neovascularization of Tregs could be associated with its immunoregulatory property, thus preserving the immune privilege of the injured cornea. Hence, the transfer of Tregs might serve as an attractive tool to improve prognosis after alkali injury.

In summary, this study is innovative in reporting the therapeutic efficacy of local delivery of Tregs in treating corneal alkali burns, by harnessing diverse mechanisms to fulfill homeostatic functions partly via the mediation of this process by Areg. It demonstrated that autologous Treg treatment had translational potential in treating human corneal alkali burns.
